# Clostridium septicum Bacteremia As the Presenting Sign of Colon Cancer

**DOI:** 10.7759/cureus.45343

**Published:** 2023-09-16

**Authors:** Andrew T Abraham, Sripal Padam

**Affiliations:** 1 Internal Medicine, University of Central Florida College of Medicine, Graduate Medical Education/Hospital Corporation of America (HCA) Florida, North Florida Hospital, Gainesville, USA

**Keywords:** colorectal cancer, infectious aortitis, tissue polypeptide specific antigen, molecular carcinogenesis, clostridium infections

## Abstract

Colon cancer is one of the leading causes of morbidity and mortality throughout the world. Some of the most common presenting signs are a change in bowel habits, alteration of fecal contour or consistency, blood in stool, fatigue, and weight loss. However, it may present insidiously. This is the case of an 81-year-old female with *Clostridium septicum* bacteremia as the primary presenting sign of metastatic colon cancer. In further literature review, we discuss the genomic associations that contribute to the severity of the disease and explore the potential links between the gut microbiome and colorectal carcinoma. This article highlights risk factor modifications and lab abnormalities that may be useful for the primary care provider and acute care practitioner.

## Introduction

*Clostridium septicum* is a common enteric bacterium that is known to have a suspected link to colon cancer. As evidenced by this case, it is tantamount that we keep a low threshold for suspicion as obvious signs of malignancy may not be initially seen. Here we will be presenting a case of an 81-year-old female with *Clostridium septicum* bacteremia as the primary presenting sign of metastatic colon cancer. Furthermore, we discuss this bacterium as well as the pathophysiology of septicemia and associated complications that may be seen. Additionally, we will review the clinical presentation of colorectal cancer (CRC), screening and diagnostic workup, and additional microbiologic associations with CRC.

## Case presentation

The patient is an 81-year-old African-American female with a pertinent history of hypertension, hyperlipidemia, type 2 diabetes mellitus, renal cell carcinoma status post right nephrectomy, and chronic kidney disease, who presented on day 1 of her hospital course to the emergency department by emergency medical services for shortness of breath and wheezing. These symptoms had been present for approximately one month and had acutely worsened two days prior to arrival. She had associated non-productive cough along with left lower quadrant pain on the abdominal exam, which she endorsed was chronic. Chest X-ray demonstrated multifocal right-sided airspace opacity, suggestive of pneumonia. Also noted was that she tested positive for COVID-19 infection on the polymerase chain reaction (PCR) test, despite being vaccinated with primary and booster immunizations. Computerized tomography (CT) of the abdomen was read as negative for any acute process at that time. She met sepsis criteria on admission, warranting blood cultures to be drawn along with fluid administration. She was on room air and did not require additional oxygen supplementation. She was started on intravenous dexamethasone, doxycycline, and ampicillin/sulbactam. Manual blood cultures demonstrated gram-variable rod bacteremia on the third hospital day. On the fifth day post-admission, speciation of blood cultures was positive for *Clostridium septicum*, and the infectious disease specialist was consulted with the recommendation to start oral metronidazole. In addition, they also advised obtaining a colonoscopy to rule out underlying colonic malignancy. She was discharged on hospital day 12 with the recommendation to obtain an endoscopy as an outpatient.

**Figure 1:**
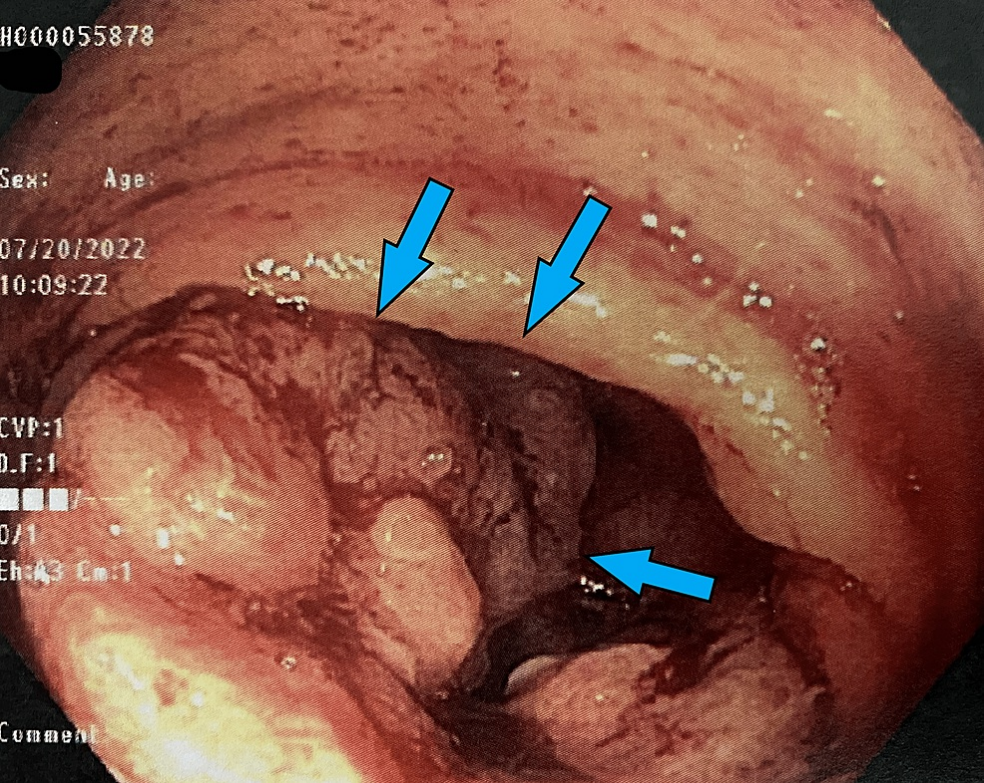
Gross view of the ascending colon on colonoscopy Large ulcerated mass (*blue arrows*) partially obstructing the lumen of the colon.

**Figure 2:**
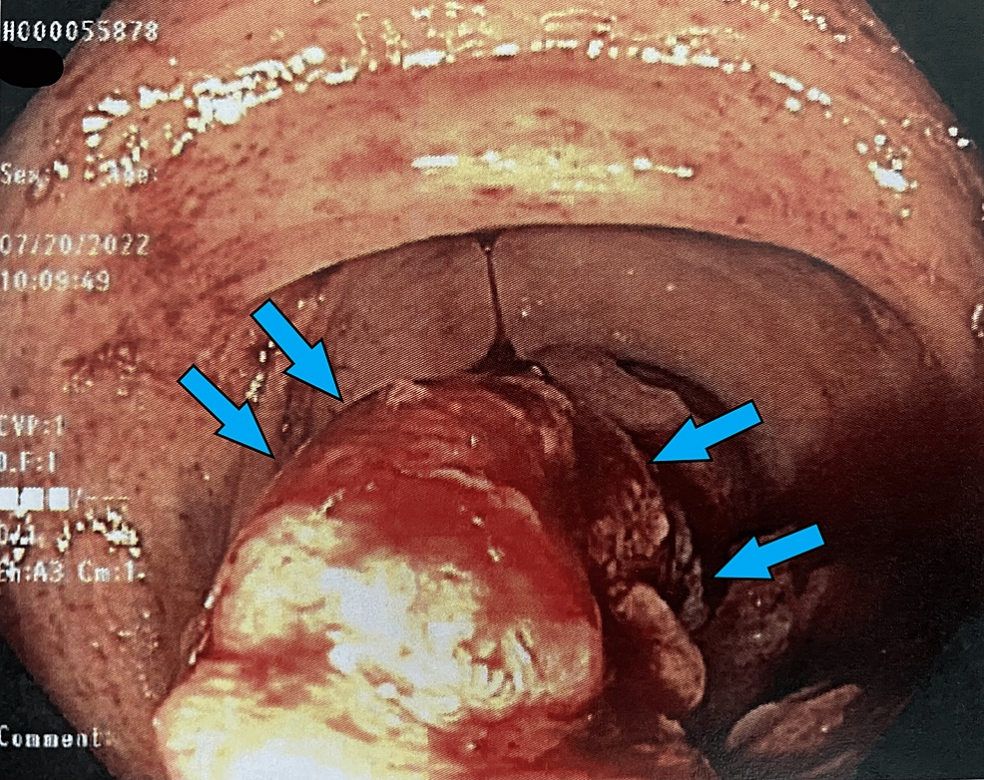
Gross view of the ascending colon on colonoscopy Large ulcerated mass *(blue arrows*) partially obstructing the lumen of the colon.

**Figure 3:**
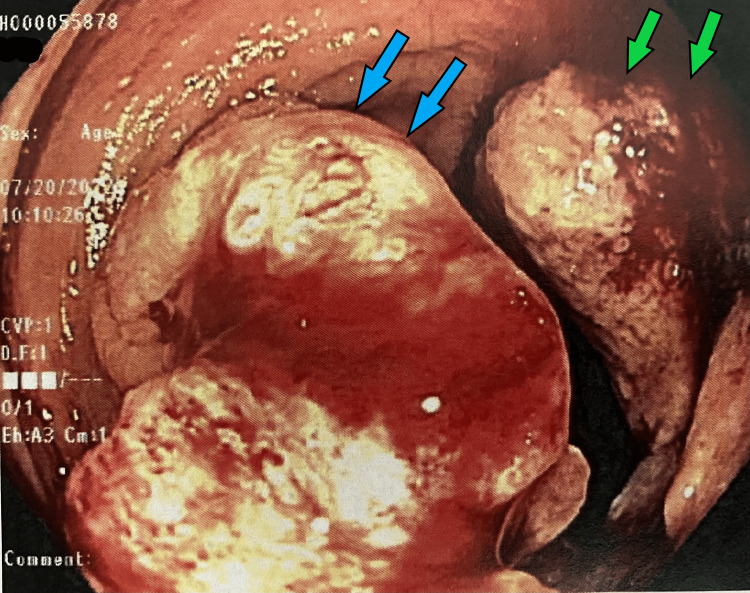
Gross view of the ascending colon on colonoscopy Two separate masses attached to the bowel wall (*blue arrows, green arrows*).

**Figure 4:**
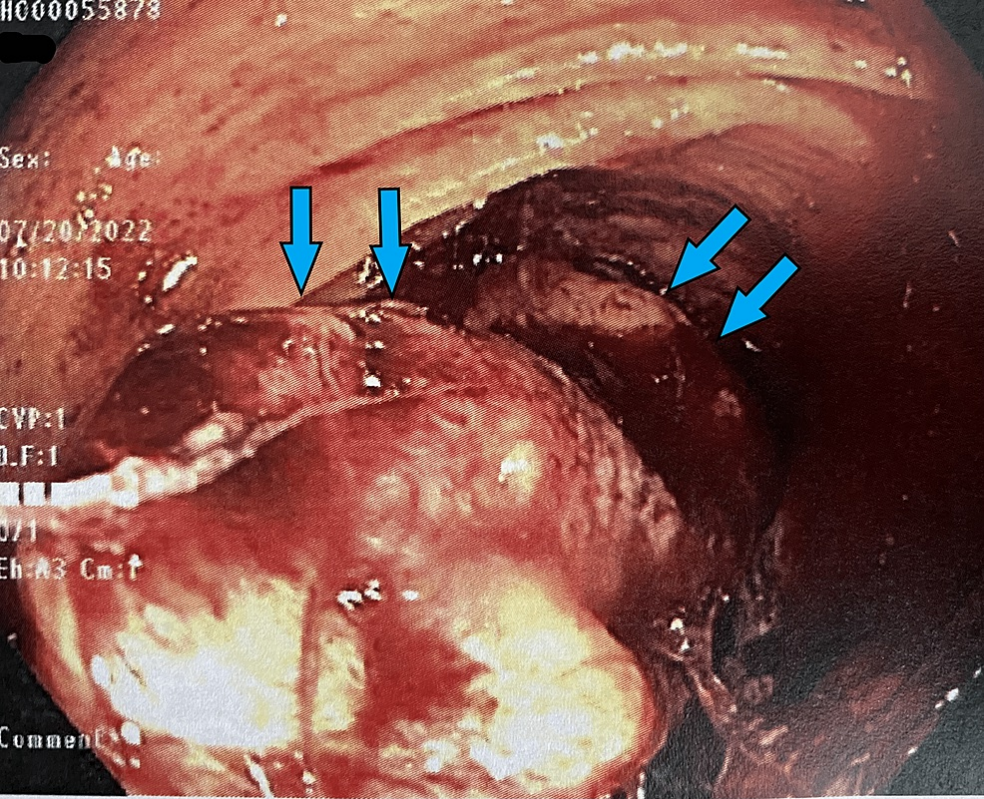
Gross view of the hepatic flexure on colonoscopy Large ulcerated mass (*blue arrows*) partially obstructing the lumen of the colon.

**Figure 5:**
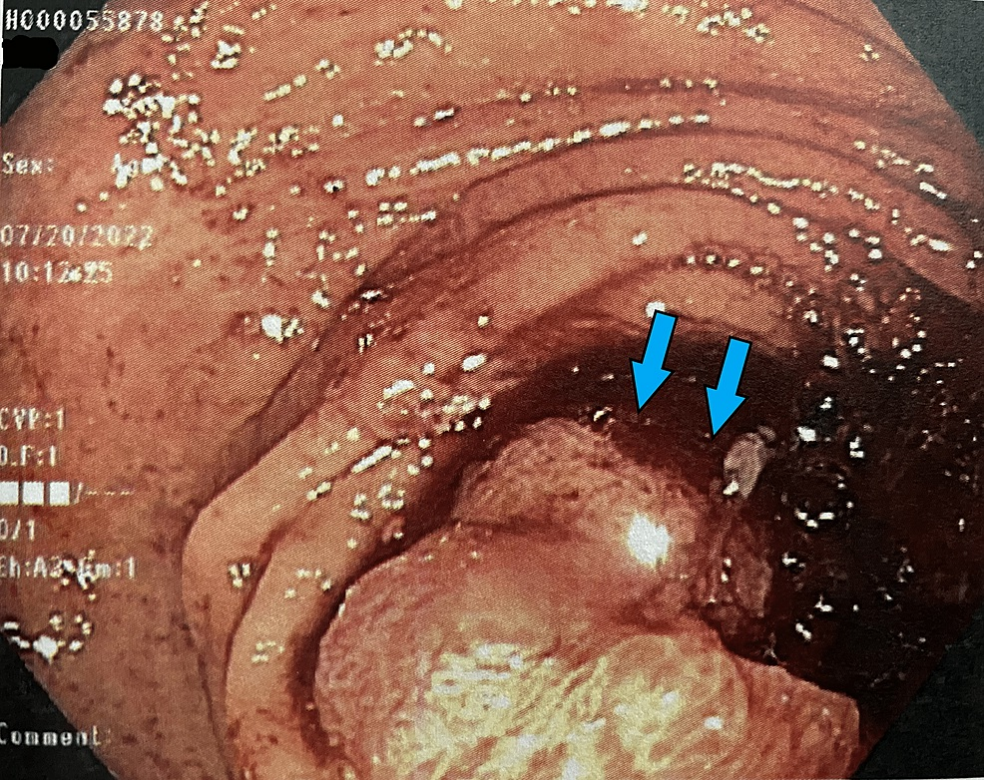
Gross view of the hepatic flexure on colonoscopy Large ulcerated mass (*blue arrows*) partially obstructing the lumen of the colon.

**Figure 6:**
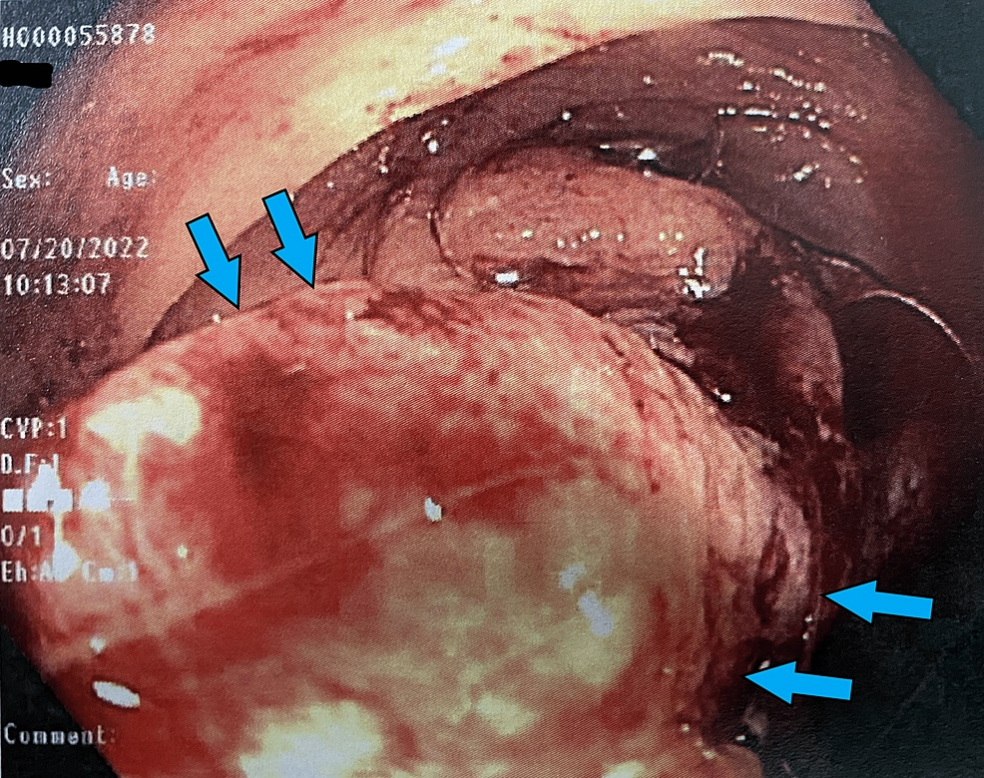
Gross view of the hepatic flexure on colonoscopy Large ulcerated mass (*blue arrows*) extending along the colonic wall.

She was seen in the outpatient setting one week after discharge and underwent a bi-directional endoscopy. Several colonic masses were identified (Figures 1-6) and biopsy results returned positive for moderately differentiated adenocarcinoma while stomach biopsies were negative for* Helicobacter pylori* on culture and negative for malignancy. In addition, carcinoembryonic antigen (CEA) was ordered and was elevated at 20.4 ng/mL (reference range is 0-2.9 ng/mL).

**Figure 7:**
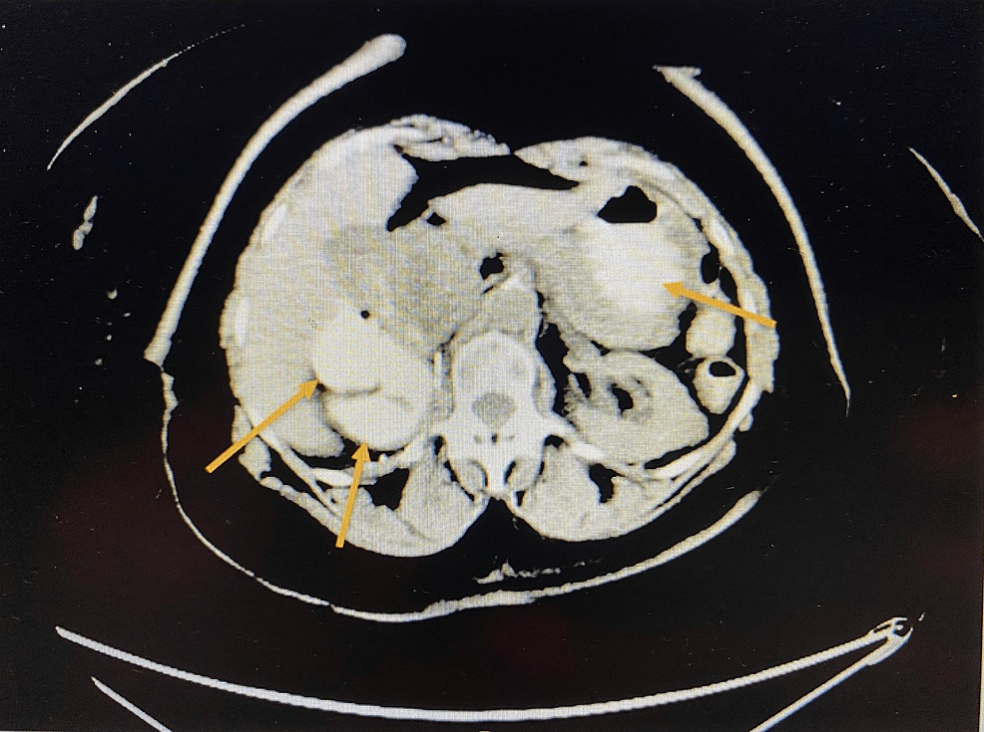
Computerized tomography scan of the abdomen after the bi-directional endoscopy showing masses in ascending and transverse colon Masses identified by yellow arrows.

CT abdomen and pelvis (Figure 7) was repeated one month after the bi-directional endoscopy which found a mass-like appearance in the ascending and transverse colon and dilation of the common bile duct and pancreatic duct and no evidence of bowel obstruction.

Magnetic resonance cholangiopancreatography was performed which revealed mild to moderate biliary ductal dilation that was traced back to the pancreatic head and ampulla. Upper endoscopy ultrasound was performed to assess the cystic pancreatic lesions, which were found to represent possible neoplasms. It should be noted that she previously had an esophagogastroduodenoscopy and colonoscopy several years prior, which revealed a large ascending colon mass and medium circumferential gastric mass in the pre-pyloric region, which were subsequently biopsied. Additionally in the past, she had multiple adenomatous polyps removed as well as presumptive benign cystic lesions throughout the pancreas. Given this, she was referred to hematology/oncology and surgical oncology and had a successful right hemicolectomy with adjunctive chemotherapy with good postoperative results.

## Discussion

In the United States and throughout the world, CRC remains one of the leading causes of cancer-related deaths. Therefore, it is imperative for early identifiers of colon cancer to be promptly identified. Several key features include rectal bleeding, weight loss, abdominal pain, and alterations in bowel patterns. In conjunction with these concerning symptoms, a positive test for fecal occult blood should immediately garner a further workup [1,2]. Several hematologic lab findings that are recognized are low hemoglobin value and low mean corpuscular volume (MCV) from baseline, usually secondary to iron deficiency anemia from occult blood loss. Additionally, an elevated red cell distribution width (RDW), which has an 84% sensitivity and 88% specificity for right-sided colon cancer, may be seen [3]. Several tumor markers may be used in the detection of CRC. One of the earliest tumor markers used for CRC detection was CEA, with the sensitivity and specificity being 65% and 90%, respectively [4]. However, elevations may be seen in a variety of other gastrointestinal conditions, making it primarily suitable for post-operative surveillance. Tissue polypeptide specific antigen (TPS) has a sensitivity and specificity of 95% and 83%, respectively [4]. Using these two tumor markers in conjunction may increase pre-test probability and suspicion of malignancy. Furthermore, hematopoietic growth factors, such as Interleukin-6, and enzymes, such as serum and urine N-acetyl-β-D-hexosaminidase and CathepsinD, have been studied for their diagnostic accuracy. However, the utility of these tumor markers for diagnosis has been limited [4]. The presence of symptoms, such as rectal bleeding or weight loss, may signify a more advanced progression of CRC. It is tantamount to outpatient screening of asymptomatic individuals being done [1,2]. With the advent of noninvasive stool tests for multiple genetic targets specific for colonic malignancy, such as K-RAS mutation and TP53, it only serves to improve detection in the general population [5]. Thus, a low threshold for suspicion of CRC must be established.

*Clostridium septicum* is a motile gram-positive spore-forming anaerobic rod-shaped bacillus of the clostridium family of toxin-producing bacterium. The primary mechanisms of virulence are via alpha toxin, beta toxin, gamma toxin, and delta toxin production. The mechanism of the produced alpha toxin is intestinal necrosis and gas gangrene resulting in translocation of the bacteria into the bloodstream. Specifically, the toxin creates epithelial pores via ionic channels, which stimulate necrotic pathways leading to cell death [6,7]. The beta toxin is another enzyme produced, which acts like a DNAse and promotes dissemination. The most common presenting symptoms are fever, chills, abdominal pain, and bowel habit disruptions, however, more severe presentations such as cellulitis, myonecrosis, abscess formation, and septic shock may occur [8]. Blood cultures that reveal *Clostridium septicum* are typically followed by prompt treatment with IV antibiotics. The antibiotics of choice are IV piperacillin/tazobactam 4.5 g every 6 hours and IV metronidazole 500 mg every 8 hours [9]. Surgical debridement is often necessary if intestinal myonecrosis is suspected. After acute management of infection, further investigation should be strongly considered to assess for malignancy.

There are several complications associated with *Clostridium septicum* bacteremia that must be identified, one of those being aortitis. The primary mechanism by which this occurs is by compromise of the intestinal mucosa and subsequent seeding of *Clostridium septicum* bacteria through the intestinal wall. From there, the gas-forming bacteria infiltrate the tunica media of the vascular wall. Chronic atherosclerosis makes the severity of *Clostridium septicum* aortitis much higher and further complications, such as mycotic aneurysm and aortic dissection, may develop. Although the overall incidence of this is exceptionally rare, it is fatal without surgical intervention [10]. The spectrum of complications is wide and other presentations that may be seen are soft tissue gas gangrene, endocarditis, brain abscess, septic arthritis, and cyclical neutropenia [11,12]. While *Clostridium septicum* bacteremia has a known association with colon cancer, this course may also be seen in hematological malignancy. In particular, several cases of sepsis from *Clostridium septicum* in patients with acute myeloid leukemia have been identified, and despite aggressive management in those cases, the prognosis was poor and inevitably led to death [13,14]. Hence, it is crucial to identify *Clostridium septicum* bacteremia early to reduce the risk of further complications to the patient.

The link between *Clostridium septicum* and colorectal malignancy is well observed in the literature. *Clostridium septicum* thrives in the gut due to its anaerobic and spore-forming properties. It directly penetrates the mucosa and spills into the bloodstream [7]. However, the pathophysiology behind how it promotes colonic malignancy is not fully understood. A recent study identified the UshA genotoxin in *Escherichia coli *led to direct DNA damage and defective cellular repair in the colon triggering carcinogenesis in mice [15]. In addition to the direct effects of infectious agents on the cellular level, diet, genetics, environment, and comorbid conditions, such as diabetes, may magnify these molecular alterations in the gut. *Clostridium septicum* may follow a similar pathogenesis, however, this has not been established. Antibiotics, like erythromycin, metronidazole, and cefoxitin, have been shown to successfully reduce intestinal bacterial load that promotes carcinogenesis, and therefore considered prophylactic agents. Therefore, the identification of a microbiologic etiology with blood cultures and PCR technology could serve as a useful biomarker and may hold the key to screening, diagnosis, and therapies [16].

There are additional infectious agents that have a strong association with colon cancer. *Streptococcus bovis*, a group D streptococcus, is a familiar association to many clinicians. It is part of the normal gastrointestinal flora and most noteworthy can cause endocarditis with bloodstream infection. *Human polyomavirus 2*, colloquially known as JC virus, is a common childhood infection that normally remains dormant within the kidneys of normal individuals. However, immunosuppression can lead to the reactivation of the virus and cause a progressive multifocal leukoencephalopathy (PML), a devastating central nervous system disease. Additionally, there have been several studies that have demonstrated high levels of *Human polyomavirus 2 *within biopsy-confirmed neoplastic tissue of the colon. This is noteworthy, as this virus contains a protein that binds to p53, a critical cell cycle checkpoint gene that is often inhibited in CRC.* Helicobacter pylori* infection has a strong association with gastric carcinoma due to its pro-oncogenic protein, however, a recent meta-analysis illustrated a correlation to CRC. It is suspected that the same pathogenesis occurs in the colonic tissue or it could possibly be a byproduct of increased gastrin secretion. Lastly, Human papillomavirus (HPV), which also has a strong carcinogenic link to cervical cancer, may also be linked to CRC [12,17]. Furthermore, it has been hypothesized that alterations to the intestinal microbiome may play a pivotal role in the enhancement of CRC risk. This is due to the alterations of the finely tuned balance of pro-inflammatory and anti-inflammatory gut flora that maintain mucosal integrity and regulate digestion [18]. Recognizing these links between infectious etiologies with colorectal carcinogenesis may guide us in our detection of CRC as well as discovering effective therapies that augment our intestinal microbiome.

## Conclusions

*Clostridium septicum *is a gram-positive bacterium that is highly associated with colonic malignancy. In acute infection, patients may exhibit nonspecific symptoms such as fever and abdominal pain. More severe complications that could be seen are cellulitis, intestinal necrosis, and septic shock which may lead to death. Early identification with blood cultures is critical and early administration of IV antibiotics should not be delayed. It is also imperative to have a low threshold of suspecting colon cancer as an underlying complication and further investigation must be done. Furthermore, the advent of new tumor markers and the identification of other carcinogenic pathogens may provide more evidence of malignancy in allow for additional treatment options. Further research is required to investigate the connection between microbiological agents and carcinogenesis.
